# Sea buckthorn bioactive metabolites and their pharmacological potential in digestive diseases

**DOI:** 10.3389/fphar.2025.1637676

**Published:** 2025-09-23

**Authors:** WenChang Dong, YuChen Tang, JiaLe Qiao, ZhiQiang Dong, Jie Cheng

**Affiliations:** ^1^ Department of Clinical Pharmacy Laboratory, The First Affiliated Hospital of Baotou Medical College, Baotou, China; ^2^ School of Pharmacy, The University of Sydney, Sydney, NSW, Australia; ^3^ School of Pharmacy, Key Laboratory of Molecular Pharmacology and Drug Evaluation (Yantai University), Ministry of Education, Collaborative Innovation Center of Advanced Drug Delivery System and Biotech Drugs in Universities of Shandong, Yantai University, Yantai, China

**Keywords:** sea buckthorn, digest disease, bioactive metabolites, clinical trials, pharmacological mechanisms

## Abstract

Sea buckthorn is a botanical drug with a long history of medicinal use in treating digestive diseases. It is considered “a food with medicinal and edible homology”, meaning it has various application scenarios. Sea buckthorn is known to have numerous bioactivities, such as anti-inflammatory, flora-regulating, immunoregulating, intestinal protective, and anticancer properties, as a potential natural therapy for digestive diseases. In both *in vitro* and *in vivo* experiments, ranging from cell lines to animal models and human patients, sea buckthorn has shown beneficial effects on symptoms associated with digestive disease. This study reviews the main bioactive metabolites of sea buckthorn and discusses their pharmacological effects and mechanisms in treating digestive diseases. In particular, we highlight bioactive metabolites isolated from sea buckthorn, their effects on inflammation, cancer, anti-*Helicobacter pylori*, radiation, and gut microbiota, and their molecular mechanisms of action in clinical applications. This article provides insight into the benefits of sea buckthorn, encouraging academic research in this area and the expansion of sea buckthorn-based applications for digestive diseases.

## 1 Introduction

**FIGURE 1:**
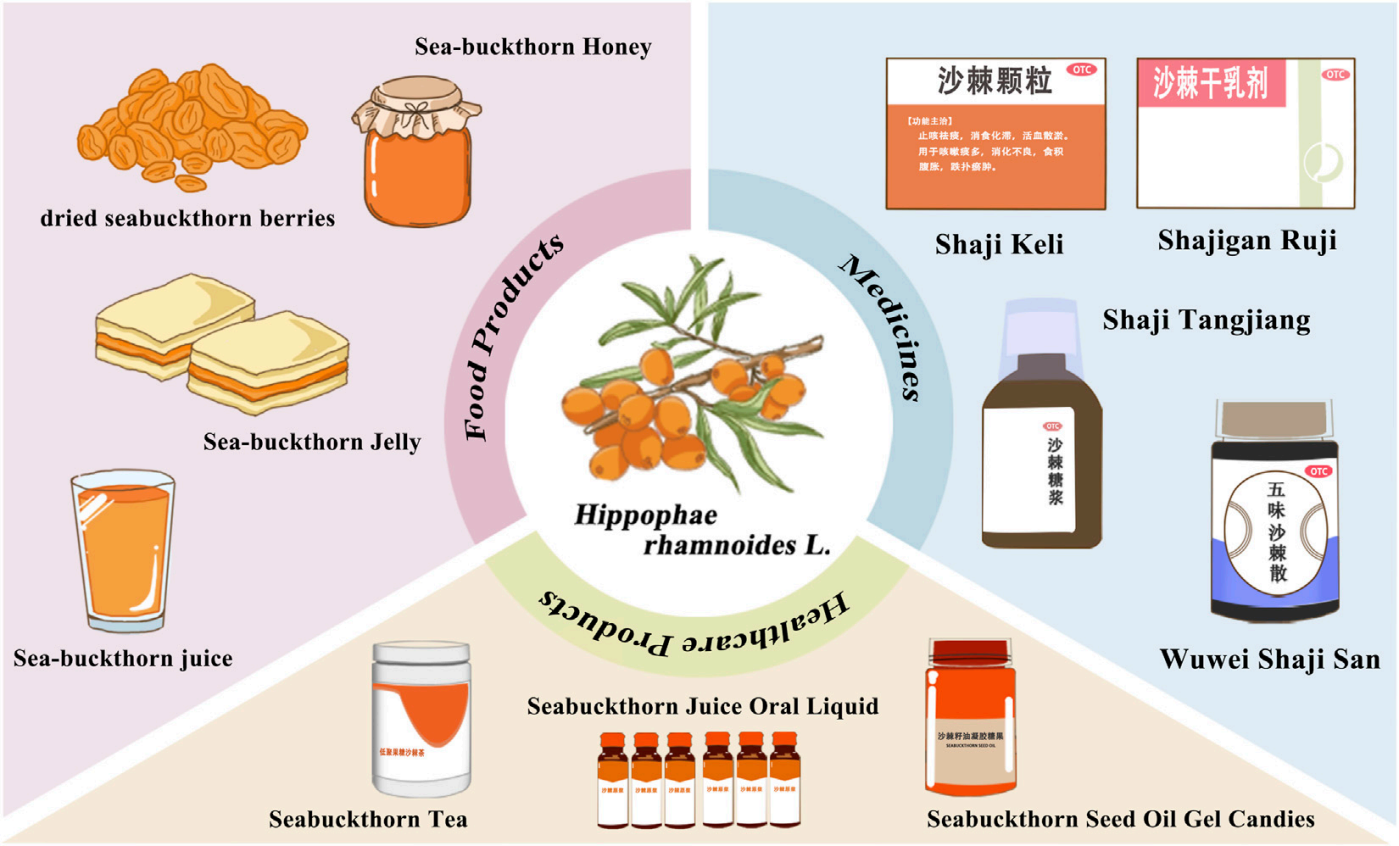
Sea buckthorn plant, medicine, food.

Sea buckthorn (*Hippophae rhamnoides* L.) is a deciduous shrub or small tree belonging to the family *Elaeagnaceae* and is known for its inducible rooting characteristics (Olas, 2018). The species grows widely in temperate, cold temperate, and subalpine regions of the Eurasian continent and is also widely cultivated in countries such as China (Singh, 2022). There are six species and eight subspecies in China, named the “Kingdom of Sea Buckthorn” (Mei et al., 2023). Due to its ability to thrive in the harshest environments, it is widespread in northwestern, northeastern, and Inner Mongolian China. In addition, its high tolerance to salt and drought can help improve the soil and prevent land degradation, which is essential for local ecology and economic development (Wang et al., 2019). In 2002, the National Health Commission of China classified sea buckthorn as a food with both medicinal and edible homology (Teng et al., 2024). Sea buckthorn has been extensively developed into functional foods and dietary supplements worldwide due to its pleasant taste and many health benefits, such as antioxidant, anti-radiation, and sea buckthorn effects (Figure 1). Sea buckthorn has gained increasing attention recently as a “Gold Bush” with significant economic, ecological, medicinal, and food value due to its diverse pharmacological and nutritional functions.

The digestive system, which encompasses the gastrointestinal (GI) tract, liver, pancreas, and gallbladder, facilitates the breakdown of food into absorbable nutrients. Digestive diseases encompass a broad range of conditions that affect the gastrointestinal tract, including gastroenteritis, precancerous gastric lesions, hepatitis, liver fibrosis, digestive cancer, and other chronic diseases (Smyth et al., 2020; Li et al., 2024; Liu et al., 2024; Mehta et al., 2024). In 2019, the global incidence of digestive diseases was considerable, with an age-standardized incidence rate of 95,582 per 100,000 person-years across 204 countries and territories (Wang Y. et al., 2023). The findings indicate that digestive diseases contribute significantly to the global healthcare burden, with over one-third of all cases having a digestive etiology worldwide (Wang F. et al., 2023). Current treatments for digestive diseases include GI surgery, gastric mucosal protective agents, antibacterial agents, and other therapeutic agents. However, these treatments often require long-term use and are associated with high recurrence rates, invasion, and adverse effects. In recent years, with the emergence of integrated traditional Chinese and Western medicine, many studies have shown that botanical drug has the following characteristics: stable pharmacological effects, high safety, and low drug resistance (Chen L. et al., 2023). Sea buckthorn contains nearly 200 known bioactive metabolites, including polysaccharides, flavonoids, vitamins, polyphenols, fatty acids, and phenolic metabolites. It has been used in traditional Chinese medicine since the Tang Dynasty, dating back over 1000 years (Suryakumar and Gupta, 2011; Li C. et al., 2018; Żuchowski et al., 2020; Lee et al., 2021). Tibetans used sea buckthorn as a medicine to treat lung and stomach diseases, and Mongolians used sea buckthorn as a sacred food, calling it “the emperor’s painstaking efforts” for medical treatment and food (Niesteruk et al., 2013; Pundir et al., 2021). Modern pharmacological studies have demonstrated that sea buckthorn has anti-inflammatory, anticancer, and digestive system regulatory properties in both animal and human *in vivo* studies (Tkacz et al., 2019; Masoodi et al., 2020; Geng et al., 2022; Qin Q. et al., 2024). Sea buckthorn is a valuable tool for preventing and treating digestive diseases.

However, in the existing and available literature, no comprehensive reviews focus solely on sea buckthorn for treating digestive diseases. Several published review articles have focused on the effectiveness of sea buckthorn in preventing and treating metabolic syndrome, radiation-induced nausea and vomiting, and its potential applications in female reproduction (Olas, 2016; Chen et al., 2023d; Mihal et al., 2023; Palatty et al., 2024). Therefore, in this article, we review recent advances in the study of natural bioactive metabolites derived from sea buckthorn and their effects on preventing and treating gastric precancerous lesions, colitis, dyspepsia, and other digestive diseases.

## 2 Literature review

### 2.1 Search strategy

We searched PubMed, Embase, Web of Science, WanFang, and CNKI databases from 1970 to March 2025. The search terms used were combined text and Medical Subject Headings (MeSH) search strategy was used to search the above databases: (“sea buckthorn” OR “Hippophae rhamnoides”) AND (“digestive” OR “liver” OR “gastric” OR “intestines” OR “pancreas” OR “gallbladder” OR “cancer” OR “tumor” OR“ gastritis” OR “enteritis” OR “gastroenteritis” OR “*Helicobacter pylori*” OR “liver fibrosis” OR “precancerous gastric lesions” OR “hepatitis” OR “inflammation”). An equivalent translation of the same search terms was used to search Chinese databases. We considered only original research and excluded reviews, surveys, conference abstracts, and editorials. This study adhered to the guidelines outlined by the Preferred Reporting Items for Systematic Reviews and Meta-Analyses (PRISMA) (Page et al., 2021b; Page et al., 2021a)

### 2.2 Inclusion and exclusion criteria

Among all studies describing associations between sea buckthorn and digestive diseases, we applied the following eligibility criteria: 1) belongs to digestive system diseases (Wang Y. et al., 2023); 2) treatment drug is sea buckthorn metabolites; 3) enough details about sea buckthorn metabolites treat digestive system diseases.

### 2.3 Data collection

**FIGURE 2:**
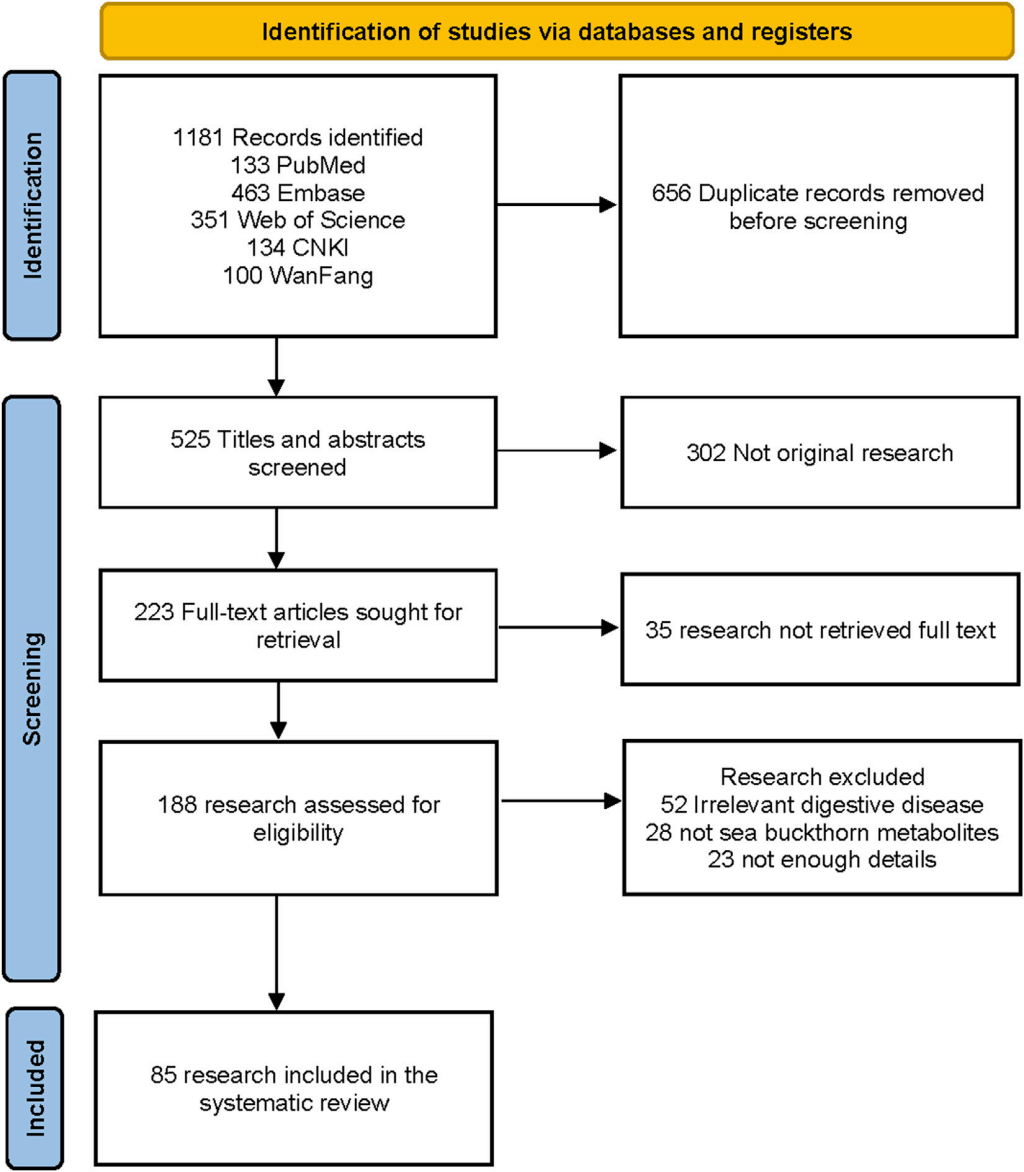
PRISMA flowchart.

Two researchers independently screened the records and extracted the data into a dedicated spreadsheet. Discrepancies between the two researchers were resolved by consensus, and if consensus could not be reached, a third reviewer was consulted. A PRISMA flow diagram was used to illustrate the literature search process and the final selection of studies (Figure 2). Data were extracted from the included studies using a standardized, predefined template. The extracted information features are summarized in Tables 1, 3–5.

**TABLE 1:** The main active metabolites in sea buckthorn for the treatment of digestive diseases.

No.	Metabolite name	Molecular formula	Type	Biological properties	Nutraceutical terminology	Ref.
1	Isorhamnetin	C_16_H_12_O_7_	Flavonoids	Antioxidative stress, anti-inflammatory, anti-cancer	Flavonoids	Lv et al. (2024), Yang et al. (2024)
2	Kaempferol	C_15_H_10_O_6_	Flavonoids	Antioxidative stress, anti-inflammatory, Gastrointestinal protective, anti-cancer	Flavonoids	Chen et al. (2023b), Zhong et al. (2024)
3	Quercetin	C_15_H_10_O_7_	Flavonoids	Antioxidant, anti-ulcer	Flavonoids	Ahmed (2024)
4	Myricetin	C_15_H_10_O_8_	Flavonoids	Anti-*Helicobacter pylori*, anti-cancer	Flavonoids	Feng et al. (2015), Krzyżek et al. (2021)
5	luteolin	C_15_H_10_O_6_	Flavonoids	Anti-inflammatory; inhibits COX-2 and iNOS in gut tissue	Flavonoids	Huang et al. (2023)
6	Apigenin	C_15_H_10_O_5_	Flavonoids	Regulate gut microbiota, anti-Helicobacter pylori, anti-cancer	Flavonoids	Kuo et al. (2014), Qiao et al. (2021)
7	Rutin	C_27_H_30_O_16_	Flavonoids	Regulate gut microbiota, anti-cancer	Flavonoids	Cai et al. (2023), Ismail et al. (2023)
8	Naringenin	C_15_H_12_O_5_	Flavonoids	Regulate gut microbiota	Flavonoids	Huang et al. (2025)
9	Naringin	C_27_H_32_O_14_	Flavonoids	Regulate gut microbiota	Flavonoids	Huang et al. (2025)
10	Hesperetin	C_16_H_14_O_6_	Flavonoids	Regulate gut microbiota, against colitis	Flavonoids	Wang et al. (2024b)
11	Catechin	C_15_H_14_O_6_	Flavonoids	Anti-cancer, regulate gut microbiota	Flavonoids	Han et al. (2021), Sun et al. (2022)
12	*p*-Coumaric acid	C_9_H_8_O_3_	Phenolics	Anti- radiation, anti-cancer	Polyphenols	Sharma et al. (2017), Li et al. (2021c)
13	Protocatechuic acid	C_7_H_6_O_4_	Phenolics	Antioxidative stress, regulate gut microbiota	Polyphenols	Crespo et al. (2017), Yang et al. (2023)
14	Ellagic acid	C_14_H_6_O_8_	Phenolics	Anti-*Helicobacter pylori*, anti-inflammatory	Polyphenols	Marín et al. (2013), De et al. (2018)
15	Palmitoleic acid	C_16_H_30_O_2_	Fatty acids	anti-inflammatory	Omega fatty acids	Shi et al. (2017), Chen et al. (2023e)
16	α-Linolenic acid	C_18_H_30_O_2_	Fatty acids	anti-inflammatory	Omega fatty acids	Shi et al. (2017), Chen et al. (2023e)
17	Ursolic acid	C_30_H_48_O_3_	Phytosterols	Anti-cancer, regulate gut microbiota, anti-inflammatory	Plant sterols	Sheng et al. (2021), Rong et al. (2024), Zhang et al. (2024b)
18	Corosolic acid	C_30_H_48_O_4_	Phytosterols	Anti-inflammatory, anti-cancer	Plant sterols	Yoo et al. (2015), Zhang et al. (2020)
19	Oleanolic acid	C_30_H_48_O_3_	Phytosterols	anti-cancer	Plant sterols	Zhou et al. (2024)
20	Lupeol	C_30_H_50_O	Phytosterols	Anti-inflammatory, Gastrointestinal protective	Plant sterols	Lee et al. (2016), Zhu et al. (2016), Somensi et al. (2020)
21	Beta-sitosterol	C_29_H_50_O	Phytosterols	Regulate gut microbiota, Gastrointestinal protective, anti-bacteria	Plant sterols	Ding et al. (2019), Ma et al. (2023), Lv et al. (2024)
22	Stigmasterol	C_29_H_48_O	Phytosterols	Antioxidative stress, anti-cancer	Plant sterols	Zhang et al. (2022), Kasprzak et al. (2023)
23	Vitamin C	C_6_H_8_O_6_	Vitamin	Anti-cancer, regulate gut microbiota, anti-inflammatory	Natural Vitamin	Kondo et al. (2019), Pham et al. (2021), Larsson et al. (2022)
24	Vitamin E	C_29_H_50_O_3_	Vitamin	Anti-inflammatory, anti- radiation	Natural Vitamin	Ahmed et al. (2023), Ganapathy et al. (2023)
25	Vitamin K_1_	C_31_H_46_O_2_	Vitamin	Anti-inflammatory	Natural Vitamin	Lai et al. (2022)
26	β-carotene	C_40_H_56_	Carotenoids	Antioxidative stress, anti-inflammatory, regulate gut microbiota, Gastrointestinal protective	Natural carotenoid complexes	Kuang et al. (2022), Wang et al. (2022)
27	Zeaxanthin	C_40_H_56_O_2_	Carotenoids	Regulate gut microbiota	Natural carotenoid complexes	Jin et al. (2024)
28	HRWP-A	—	Polysaccharide	Regulate Immunomodulatory, anti-cancer	Bioactive polysaccharides	Wang et al. (2015), Wang et al. (2018a)
29	HRP	—	Polysaccharide	Regulate Immunomodulatory, antioxidative stress, anti-cancer, anti-inflammatory, regulate gut microbiota	Bioactive polysaccharides	Zhao et al. (2019), 2020; Liu et al. (2022), Da (2023)
30	SP	—	Polysaccharide	Anti-cancer, antioxidative stress	Bioactive polysaccharides	Wei et al., 2019; Panpan (2021)

### 2.4 Study selection

Figure 2 shows the PRISMA diagram for selecting original research to be included in the analysis. The literature search resulted in 1181 records, of which 656 were duplicates and removed. We excluded 302 records that were not original study, 35 research without full text, 52 irrelevant to digestive disease, 28 not Sea buckthorn metabolites, and 23 with unclear details. The literature review included 85 research.

## 3 Traditional application of sea buckthorn on digestive diseases

### 3.1 Digestive diseases understanding in traditional Chinese medicine

The Chinese medical tradition is known for being one of the oldest and most distinctive systems of medicine in the world, with a written history stretching back nearly 3,000 years (Yu and Amri, 2016). Traditional Chinese Medicine (TCM) takes a holistic approach to health and disease, emphasizing the interconnectedness of different body systems (Zhang J. et al., 2024). In Chinese medicine, digestive diseases are attributed to imbalances within the stomach, liver, and spleen. Digestive disorders are associated with spleen-stomach deficiency syndrome, Dampness-Heat syndrome, and Liver Qi stagnation syndrome. Mongolian medicine’s systematic theoretical system is based on the balance among three roots: Heyi, Xila, and Badagan (Dao et al., 2021). When the balance is disrupted, any of these elements may experience excessive increase or depletion, resulting in loss of coordination and pathological conditions. The fundamental theory of Tibetan Medicine is an elements theory consisting of “air” “fire” and “water” (Li Q. et al., 2018). According to Tibetan medicine, the human body is connected by various parts. Balance is a crucial principle in the three systems of TCM, Tibetan, and Mongolian medicine; Digestive diseases are viewed as a consequence of imbalance.

### 3.2 Traditional approaches of sea buckthorn in digestive diseases

Sea buckthorn has been used in traditional medicine across Asia and Europe for many years (Olas, 2018). Sea buckthorn was being used as a medicinal remedy, with the earliest documentation found in the Tibetan medical classic “Somaratsa” in the first half of the eighth century and the “Medical Canon in Four Sections” describes the medicinal use of sea buckthorn (Wang, 2022). Chinese folklore treatment books record that Sea buckthorn affects the respiratory and digestive systems (Guo, 2019). For a long time, it has been used to treat slow digestion and stomach malfunction. According to “Chinese Pharmacopeia,” Sea buckthorn is characterized by acidity, astringent taste, and mild nature and belongs to the spleen, stomach, lung, and heart meridian (Chinese Pharmacopoeia Commission, 2020). It is known for its ability to promote blood circulation and disperse stasis, resolving phlegm, clearing the chest, and strengthening the spleen and stomach. The Dictionary of Traditional Chinese Medicine records that sea buckthorn has the effects of promoting fluid production and quenching thirst, clearing heat, and stopping diarrhea (Peng, 1993). According to the Tibetan medical classics “*Yue wang yao zhen*” and “*Medical Canon in Four Sections*,” sea buckthorn is characterized by strengthening the spleen and nourishing the stomach, breaking blood stasis and treating the blood-related conditions, removing phlegm and benefiting the lungs, and facilitating digestion (Suryakumar and Gupta, 2011). In Mongolian medicine, sea buckthorn is recorded as “sharp and light, which is beneficial for treating “ba da gan” of the lungs and stomach to treat colitis and enterocolitis for humans and animals (Guo, 2019; Li X. et al., 2021). In Russia, sea buckthorn is mainly used to treat gastrointestinal disorders and skin diseases (Li X. et al., 2021). Since antiquity, sea buckthorn has been a classic treatment for digestive disorders.

## 4 Sea buckthorn extracts on digestive diseases

### 4.1 Clinical use of sea buckthorn

**FIGURE 3:**
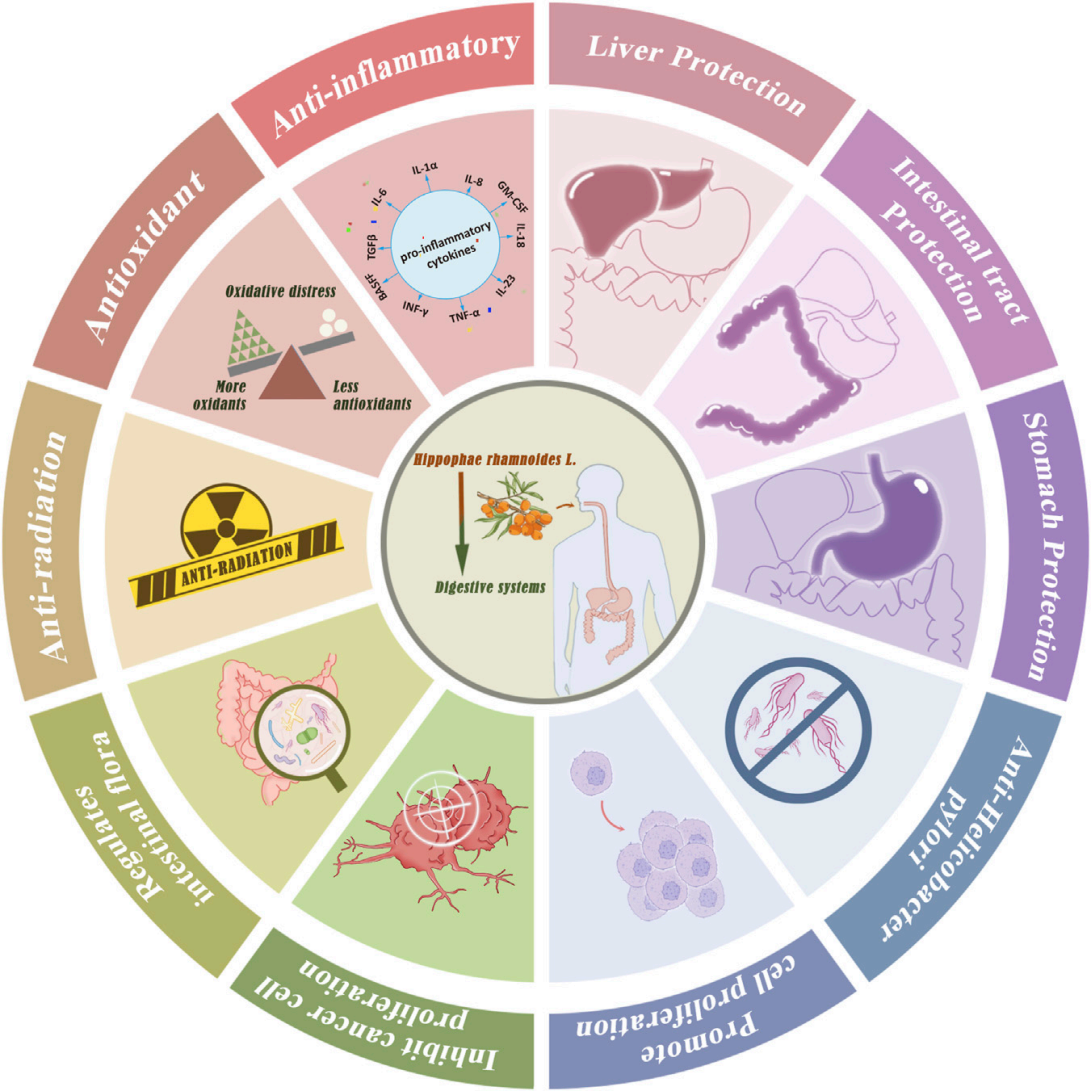
Sea buckthorn main biological activates.

Recently, sea buckthorn has attracted the attention of researchers due to its superior biological activities such as anti-tumor, hypoglycemic, immunomodulatory, and other activities (Ying, 2024). Since the 1940s, Russian scientists have been studying the bioactive metabolites in the berries, leaves, and bark of sea buckthorn. This research has contributed to the development of sea buckthorn-based foods and radiation protection creams for Russian cosmonauts (Krejcarová et al., 2015). China was the first to officially recognize sea buckthorn as a medicinal substance, including it in the Chinese Pharmacopoeia in 1977 (Chinese Pharmacopoeia Commission, 1977). Ulcerative colitis (UC) is a disease involving superficial inflammation and ulceration of the mucosal lining of the bowel. This leads to symptoms such as diarrhea, abdominal pain, and cramping (Gajendran et al., 2019). In addition, sea buckthorn polysaccharides can ameliorate intestinal barrier damage and regulate intestinal microbiota and their metabolites (Yuan et al., 2024). The above study demonstrates that sea buckthorn holds significant potential for the treatment of UC. Chronic atrophic gastritis (CAG) is recognized as a precursor to gastric cancer. Research has shown that various metabolites in sea buckthorn exhibit therapeutic effects on CAG. Sea buckthorn procyanidins have been found effective against *H. pylori*, a key factor in CAG development (Guo, 2008). Sea buckthorn oil, known for its antacid and gastric barrier properties, is used in the treatment of CAG (Yan, 2002). Additionally, sea buckthorn pulp oil has been reported to alleviate gastric discomfort and ulcers by reducing mucus production, inhibiting acid secretion, and suppressing gastric motility (Xing, 2012). Moreover, sea buckthorn extracts have been shown to treat *H. pylori*-induced gastritis by downregulating the mRNA expression of the inflammatory factors NF-κB-p65 and IκB-α (Ying, 2022). Oxidative stress is associated with numerous health issues, including cardiovascular diseases, neurodegenerative disorders, cancer, and aging, and also plays a significant role in the development of gastrointestinal diseases. Sea buckthorn leaf extract (SBLE) exhibits well antioxidant properties and has potential as a natural additive to reduce the degradation of sea buckthorn oil (SBO), as well as to provide synergistic health benefits (Lyu et al., 2022). This study confirmed that sea buckthorn berries, demonstrating biological potency through anti-α-glucosidase and anti-lipase activities, could serve as raw materials for developing innovative functional foods and nutraceuticals (Tkacz et al., 2019). Furthermore, this observational study suggests that aqueous and hydroalcoholic extracts of sea buckthorn leaves have marked cytoprotective, and antibacterial activities (Upadhyay et al., 2010). Cancer is a significant social, public health, and economic challenge in the 21st century, accounting for nearly one in six deaths (16.8%) worldwide (Bray et al., 2024). According to global cancer statistics from 2020, gastric cancer ranks fifth in incidence and fourth in mortality, posing a serious threat to human health and life (Raza and Bhatt, 2023; Bray et al., 2024). A growing number of *in vitro* and *in vivo* animal studies have confirmed the anticancer activity of sea buckthorn. Several metabolites in sea buckthorn, mainly phenolic metabolites such as procyanidins and flavonoids, have been shown to benefit cancer prevention significantly (Wang et al., 2014; Masoodi et al., 2020). Isorhamnetin, a metabolite derived from sea buckthorn, may target PI3K and block the PI3K-AKT-mTOR signaling pathway. It can significantly inhibit autophagy in gastric cancer cells under hypoxic conditions, suppress cell proliferation, reduce mitochondrial membrane potential, and promote mitochondria-mediated apoptosis (Li C. et al., 2021). Sea buckthorn procyanidins have been identified as promising inhibitors of fatty acid synthase (FAS), capable of inducing apoptosis in MDA-MB-231 cells and potentially aiding in the prevention or treatment of breast cancer (Wang et al., 2014). Sea buckthorn oil can inhibit the proliferation of human gastric cancer HGC-27 cells by activating the P53 signaling pathway and promote apoptosis, thereby exerting an anti-tumor effect (Lu-gen and Xiao-xia, 2021). A recent study suggests that sea buckthorn leaf extract may induce apoptosis and inhibit the rapid proliferation of rat C6 glioma cells (Kim et al., 2017). Chemotherapy and radiotherapy are the mainstays of cancer treatment but are associated with various side effects, including cardiotoxicity, nephrotoxicity, myelosuppression, neurotoxicity, hepatotoxicity, gastrointestinal toxicity, mucositis, and alopecia, which severely affect the quality of life of cancer patients (Liu Y.-Q. et al., 2021). Natural products have a wide chemical diversity and flexible biological properties that make them well-suited to adjuvant therapy to reduce the side effects of cancer treatment. The study indicates that Sea buckthorn extract can protect mitochondrial and genomic DNA from radiation-induced damage (Shukla et al., 2006). Polyphenols and flavonoids are thought to be responsible for scavenging free radicals and protecting DNA. In addition, sea buckthorn extract RH-3 has been shown to inhibit the Fenton reaction and radiation-mediated generation of hydroxyl radicals *in vitro*, superoxide anion-mediated nitro blue tetrazolium (NBT) reduction, and FeSO_4_-mediated lipid peroxidation in mouse liver (Goel et al., 2002). Sea buckthorn holds significant potential for the prevention and treatment of digestive diseases. However, further systematic research is needed to identify its active metabolites and clarify the underlying mechanisms. These findings support the development of the sea buckthorn industry and its future clinical applications (Figure 3).

## 5 Material basis of sea buckthorn

**FIGURE 4:**
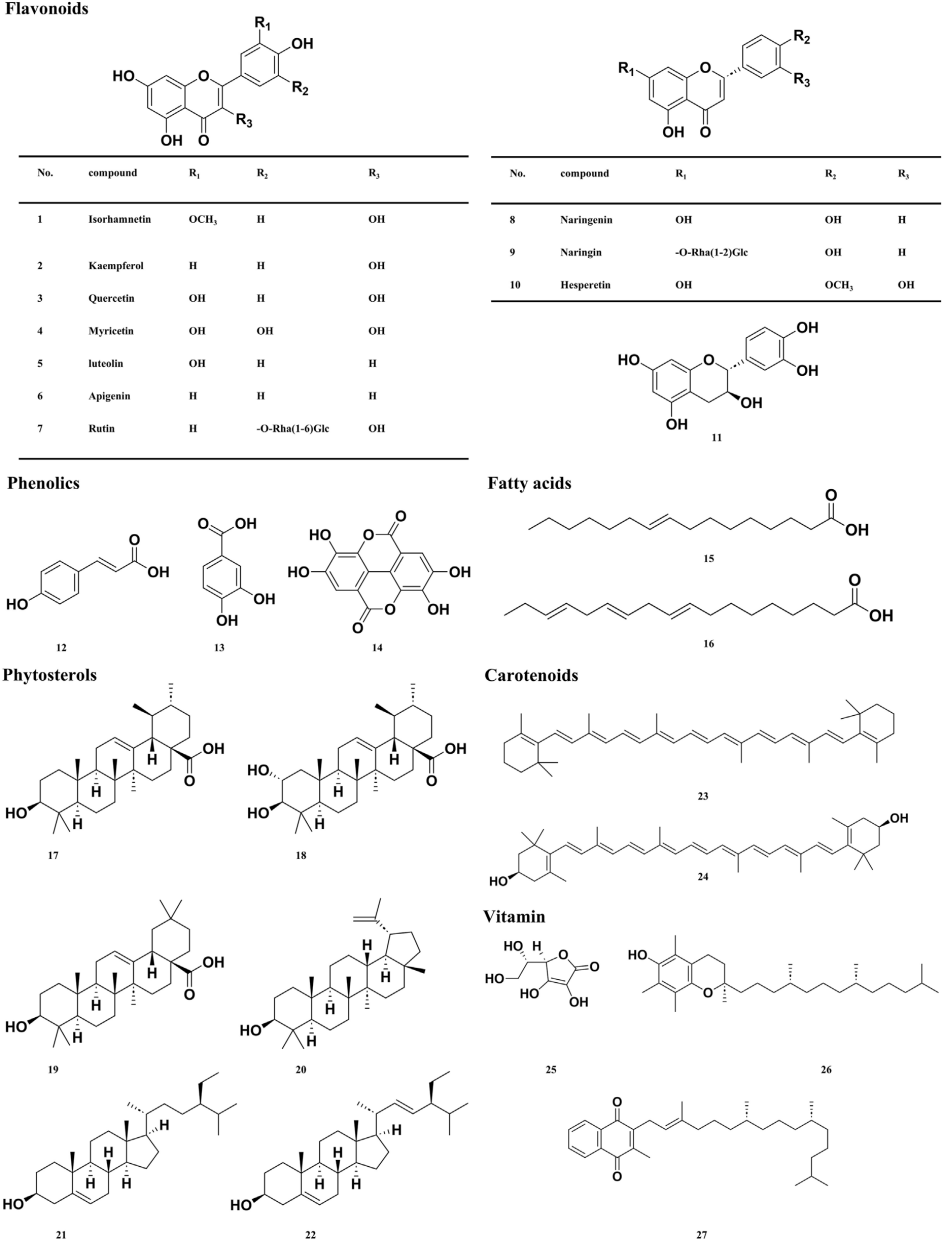
Structures of Sea buckthorn main active metabolites.

Sea buckthorn berries, seeds, and leaves have been reported to contain more than 190 bioactive metabolites, including 95 types of flavonoids (Liu S. et al., 2021; Zu Fan, 2024), 17 types of phenolic acids (Zadernowski et al., 2005), ten types of tannins (Sheichenko et al., 1987; Yoshida et al., 1991), seven types of Triterpene, 11 types of fatty acids (Yang and Kallio, 2001; Zheng et al., 2017; Zielińska and Nowak, 2017), 15 types of vitamins (Chen et al., 2023d), and 17 types of phytosterols (Li et al., 2007), 28 types of polysaccharides metabolites (Teng et al., 2024), in addition to small amounts of amino acids, organic acids, and inorganic elements. We screened the active metabolites in sea buckthorn and identified 30 metabolites that have therapeutic effects on digestive system diseases, which are listed in Table 1. The corresponding structural formulas are presented in Figure 4.

### 5.1 Flavonoids and phenolic

Over 98% of the flavonoids in sea buckthorn fruits are flavonols, with Isorhamnetin accounting for 66%–72% of the total flavonols and Quercetin making up 25%–32% of the total flavonols (Tkacz et al., 2020). Flavonoids are essential bioactive in sea buckthorns and have antioxidant and anti-inflammatory properties. They could modulate T cell differentiation, alter gut microbiota, and modulate cytokines. Sea buckthorn flavonoids extract can regulate the TAK1/p38MAPK/p65NF-κB pathway to effectively ameliorate liver injury in mice with alcoholic fatty liver disease (AFLD) and regulate the composition of the gut microbiota (Zhao et al., 2022). Plant phenolic acids are an essential metabolite of the human diet and exhibit tremendous antioxidant properties, which could significantly reduce the risk of many oxidative stress-related diseases, such as cancer. Phenolic acids treat inflammatory bowel disease by improving the barrier function of the intestinal mucosa, reducing oxidative stress, inhibiting excessive activation of the immune response, and regulating the balance of the intestinal microbiota (Lu and Han, 2024). In plant chemistry, tannins are an important subgroup of phenolic metabolites. Tannins are commonly found in the human diet and are beneficial for health; they are prevalent in plant foods, particularly in fruits, nuts, and vegetables. Tannings’ anticarcinogenic and antimutagenic potential may be attributed to their antioxidant properties, which help protect against cellular oxidative damage, including lipid peroxidation (Chung et al., 1998). We summarize the material basis of Sea buckthorn in preventing and treating digestive diseases, and the relevant details are shown in Table 1.

### 5.2 Fatty acids, carotenoids and phytosterols

Fatty acids are crucial metabolites of the human diet, and their biological activities influence the metabolism, function, and responsiveness of cells and tissues to hormonal and other signals. Fatty acids are a primary energy source and signaling molecules, affecting the gut microbiota and immune responses. Palmitoleic acid (PLA) is the primary metabolite of sea buckthorn pulp oil, while alpha-linolenic acid (ALA) is the main metabolite of sea buckthorn seed oil. Pretreatment with PLA and ALA prolonged survival time after radiation-induced acute intestinal injury (Shi et al., 2017). The dietary palmitoleic acid enhanced gut mucosal barriers, reduced inflammatory cell infiltration and the expression of TNF-α and IL-6, and improved the pharmacological effects of anti-TNF-α therapy in both acute and chronic inflammatory bowel diseases (IBD) mouse models. β-carotene could help protect against food allergies by enhancing intestinal epithelial barrier function and regulating gut microflora (Kuang et al., 2022; Wang et al., 2022). Zeaxanthin increased the abundance of probiotics and decreased the abundance of pathogens, thereby improving the dysbiosis of enteric microbial communities and enhancing the structure and diversity of the gastrointestinal microbiome in mice with obesity caused by excessive fat consumption (Jin et al., 2024). Phytosterols are naturally occurring bioactive metabolites in plants that protect against various chronic diseases, including liver disorders, diabetes, and cancer. Studies have shown that a diet rich in phytosterols may reduce cancer risk by up to 20% (Suryamani et al., 2022).

### 5.3 Vitamin and polysaccharides

Sea buckthorn is rich in various vitamins, especially vitamin C, and has been called the “King of VC”. In addition, sea buckthorn berries contain vitamin A, vitamin E, riboflavin, niacin, pantothenic acid, vitamin B6, and vitamin B_12_ (Wang, 2022). Recent studies have shown that sea buckthorn polysaccharides provide significant benefits for gut health, including the reduction of cell death and lower levels of reactive oxygen species (ROS) in the intestine (Shen et al., 2021). Additionally, *in vitro* antioxidant studies have demonstrated that sea buckthorn polysaccharides effectively scavenge superoxide anions and DPPH radicals, particularly ABTS radicals (Wang H. et al., 2024).

### 5.4 Differences in active metabolites between *Hippophae rhamnoides* L. Subspecies

The *H. rhamnoides* L. (Elaeagnaceae) comprises eight accepted subspecies (subsp): subsp. *carpatica* Rousi, subsp. *caucasica* Rousi, subsp. *mongolica* Rousi, subsp. *rhamnoides*, subsp. *wolongensis* Y.S.Lian, K.Sun and X.L.Chen, subsp. *turkestanica* Rousi, subsp. *yunnanensis* Rousi (Hippophae rhamnoides, 2025). The active metabolites in sea buckthorn vary among different subspecies, as illustrated in Table 2. Significantly, the total amounts of phenolics in the fruits of subsp.*rhamnoides* and subsp.*caucasica* differ from each other. Specifically, the total phenolics content is 51.23 ± 1.38 mg/g compared to 6.22 ± 0.3 mg/g. Extracellular antioxidant properties is closely linked to total phenols and flavonoids in the extract, whereas cellular antioxidant properties and antiproliferative effects on HepG2 cells are significantly associated with total phenolic acids and flavonoid aglycones (Guo et al., 2017b). It suggests that the subsp. *yunnanensis* Rousi has the highest phytochemical content (total flavonoids: 47.7 ± 3.6 mg/g, total phenolics: 33.2 ± 2.1 mg/g), along with significant antioxidant and antiproliferative effects. In comparison to the *mongolica* Rousi, and *rhamnoides*, the subsp.*carpatica* Rousi has the highest overall fatty acid content. Research has shown that the fatty acids in sea buckthorn have anti-inflammatory properties that help protect the mucosa of the digestive tract (Shi et al., 2017). The subsp.*yunnanensis* maximizes vitamin C content, making it rich in antioxidants and possessing anti-inflammatory properties (Guo et al., 2017b). The subspecies *wolongensis* is a newly identified subspecies found in the transitional zone between the eastern edge of the Tibetan Plateau in China and the Sichuan Basin (LL, 2015). This subspecies has a lower total flavonoid content and a higher total phenolic content compared to other subspecies. The cool, humid, high-altitude environments where this species predominantly occurs are likely more conducive to the accumulation of phenolic acids than flavonoids. The levels of metabolites in sea buckthorn vary between subspecies due to their origins and the conditions in which they grow. In contrast, the subspecies mongolica often exhibit higher total flavonoid content. This is due to their adaptation to stronger ultraviolet radiation and drier environments, where flavonoids act as protectants against UV rays and serve as antioxidants.

**TABLE 2:** Differences in composition between species.

Species	Total phenolics (mg/g)	Total flavonoids (mg/g)	Total fatty acids (%)	Total carotenoids (mg/100 g)	Total phytosterols (mg/kg)	Vitamin C (mg/100 g)	Total polysaccharides (g/100 mL)	Ref.
*Hippophae rhamnoides* subsp. *yunnanensis* Rousi	33.2 ± 2.1	47.7 ± 3.6	—	—	—	1129.1	—	Guo et al. (2017b)
*Hippophae rhamnoides* subsp. *mongolica* Rousi	30.9 ± 2.4	44.4 ± 3.2	5.9	—	—	394.9	7.7	Kallio et al., 2002; Yang (2009), Guo et al. (2017b)
*Hippophae rhamnoides* subsp. *turkestanica* Rousi	27.6 ± 1.9	34.9 ± 1.2	—	—	—	472.9	—	Guo et al. (2017b)
*Hippophae rhamnoides* subsp. *caucasica* Rousi	6.22 ± 0.3	—	—	—	—	62.85 ± 5.4	—	Ilhan et al. (2021)
*Hippophae rhamnoides* subsp. *rhamnoides*	51.23 ± 1.38	—	3.5	18.5	385 ± 60	1117.84	1.7	Andersson et al. (2009), Yang, 2009; Khan et al. (2017)
*Hippophae rhamnoides* subsp. *carpatica* Rousi	18.97 ± 0.09	—	6.2	96.7 ± 6.5	—	—	—	Dulf, 2012; Pop et al. (2014), Petrescu et al. (2022)
*Hippophae rhamnoides* subsp. *wolongensis* Y.S.Lian, K.Sun and X.L.Chen	38.8∼38.8	17.6∼27.4	—	—	—	—	—	Ll (2015)

## 6 Modern industrial development

Sea buckthorn is widely used in food, nutraceuticals, and plant-based medicines worldwide, renowned for its medicinal properties and rich nutritional benefits. The global sea buckthorn market size was valued at USD 347.56 million in 2023 and is projected to grow from USD 381.40 million in 2024 to USD 837.26 million by 2032 (Sea Buckthorn Market Size, 2024). Today, sea buckthorn is cultivated in approximately 40 countries, covering a global production area of about 3 million hectares (Nayik and Gull, 2020). China, Russia, Canada, Mongolia, and Northern Europe account for almost 90% of the world’s sea buckthorn production. China is the leading producer of sea buckthorn globally, with over 10 million acres cultivated artificially and an additional 8 million acres in the wild. The processing and utilization of sea buckthorn fruit amounts to 80∼100 thousand tons annually, contributing to a total annual output value of 3.3–3.6 billion dollars in various sea buckthorn industries (Sea Buckthorn Professional Committee of China Society of Sand Control and Sand Industry, 2024). However, most sea buckthorns are insufficiently exploited, with a single-product structure and low value added (Yi, 2023). Various finished products have emerged with the development of modern sea buckthorn processing technologies (Zhang et al., 2023). To strengthen the sea buckthorn industry, improvements in the production system are essential. Innovative processing technologies must be developed, public awareness of sea buckthorn products needs to be increased, and its uses in food and medicine should be further promoted. In the context of food applications, sea buckthorn’s medicinal and nutritional properties—such as promoting digestion, relieving cough, and reducing phlegm, as recorded in the Chinese Pharmacopoeia (Chinese Pharmacopoeia Commission, 2020), make it a valuable metabolite for the development of various functional food products. These include breads, yogurts, jams, beverages, teas, and other formulations (Selvamuthukumaran and Khanum, 2014; Ghendov-Mosanu et al., 2020; Gâtlan and Gutt, 2021), which have been shown to stimulate appetite, boost energy levels (Chen A. et al., 2023), and enhance immune function (Dubey et al., 2023). During the COVID-19 pandemic, sea buckthorn was found to boost immunity and anti-coronavirus (Al Ibrahim et al., 2023). In the field of daily chemical products, the anti-ultraviolet, wound healing, anti-aging, and antioxidant properties of sea buckthorn are used to make cosmeceuticals, emulsions, and essential oils to protect the skin from the sun and repair skin damage (Koskovac et al., 2017; Zosimidou et al., 2023; Okamoto et al., 2024). In medicine, the bioactive metabolites in sea buckthorn are extracted to treat gastritis, indigestion, diabetes, cancer, stroke, and cardiovascular disease (Xu et al., 2011; Olas et al., 2018; Shen et al., 2021). For this reason, the development and application of sea buckthorn have significant medicinal and economic value.

## 7 Clinical studies

Several clinical controlled trials have shown that sea buckthorn, sea buckthorn extract, or sea buckthorn-related combination therapy can be beneficial in preventing and treating digestive diseases. Digestive diseases, including nonalcoholic fatty liver disease (NAFLD), viral diarrhea, chronic gastritis, and functional dyspepsia, significantly increase the economic burden of digestive diseases globally (Wang Y. et al., 2023). A large meta-analysis involving 9275 patients from Taiwan found that habitual cigarette smoking, alcohol consumption, and betel chewing were associated with a 16.32-fold risk of esophageal cancer (Chuang et al., 2017). This highlights that an unhealthy diet plays a major role as a risk factor for developing digestive diseases, and considering that sea buckthorn is a great dietary supplement, it has great potential in preventing and treating digestive diseases. Digestive diseases are interrelated, necessitating a holistic approach for both prevention and treatment. We summarized eight relevant clinical studies involving 513 patients to clarify the clinical effects of sea buckthorn against digestive diseases. A study conducted on patients with liver fibrosis showed that sea buckthorn extract has anti-inflammatory effects that can reduce the level of inflammation in the body, reduce TNF-α, IL-6, total bile acid (TBA) concentration and significantly shortens the time for normalization of aminotransferases, thus sea buckthorn extract may be a hopeful drug for prevention and treatment of liver fibrosis (Gao et al., 2003). In addition, a study was conducted on people with NAFLD, and the results suggested that sea buckthorn capsules can significantly decrease the serum levels of alanine aminotransferase (ALT), LDL-C, hyaluronic acid, collagen type IV and CT liver/spleen ratio, which may be further developed as a promising therapy for the treatment of NAFLD (Gao et al., 2014). One study showed that sea buckthorn can reduce the concentration of C-reactive protein (CRP), thereby reducing the risk of inflammation and cardiovascular diseases (Larmo et al., 2008). Two studies found that sea buckthorn emulsion may promote gastrointestinal motility and relieve symptoms of dyspepsia (Shi and Xuan, 2020; Ting and Ping, 2023). Additionally, a study found that sea buckthorn could reduce the clinical symptoms of chronic gastritis, increase appetite, repair the stomach lining, reduce and eliminate *H. pylori*, and increase motilin levels (Feng, 2015). In conclusion, sea buckthorn has demonstrated significant pharmacological effects in improving digestive symptoms, reducing the risk of liver damage, and treating functional dyspepsia. Table 3 provides more details on the clinical trials. While some studies used randomized designs, inadequate blinding procedures may have biased outcome assessments (Gao et al., 2014). Because there are few clinical studies on sea buckthorn for treating digestive system diseases, it is challenging to extract high-quality clinical trial evidence from them.

**TABLE 3:** In clinical studies of sea buckthorn treatment of digestive diseases.

Study design	Study subject	Sea buckthorn group (n)	Control group (n)	Effect	Mechanism	Ref.
Randomized, controlled	Liver fibrosis (n = 50)	Extract (15 g tid), 6 months (n = 25)	Positive group: vitamin B (1 tablets tid), 6 months (n = 25)	Prevention and treatment of liver fibrosis	TBA↓, laminin, hyaluronic acid↓, collagen types III and IV↓	Gao et al. (2003)
Randomized, placebo-controlled	NAFLD	Capsules (1.5 g tid), 90 days (n = 48)	Negative group: placebo capsules (1.5 g bid), 90 days (n = 46)	Promising therapy for the treatment of NAFLD	Liver/spleen ratio↓, hyaluronic acid↓, collagen type IV↓	Gao et al. (2014)
Randomized, double-blind, randomized, placebo-controlled	Healthy volunteers	Extract (28 g qd), 90 days (n = 116)	Negative group: placebo (28 g qd), 90 days (n = 117)	Reduce infections symptoms	CRP↓	Larmo et al. (2008)
Randomized, controlled	Children viral diarrhea	Emulsion plus interferon α-1b; emulsion (<1 year: 2.5 g bid; >1 year: 5 g bid), 5 days; α-1b (<1 year: 6 μ g qd; >1 year: 10 μ g qd), 5 days (n = 44)	Positive group: montmorillonite powder (≤2 years: 2 g tid; >2 years: 3 g tid), 5 days (n = 44)	Improve clinical symptoms and intestinal flora	Dehydration correction time↓, diarrhea off-time↓, hospitalization time↓	Su and Hong (2022)
Randomized, controlled	Chronic gastritis	Emulsion (20 g tid), 1 month (n = 43)	Positive group: xiang sha yang wei pills (10 pills tid), 1 month (n = 43)	Improve clinical symptoms, increase appetite, repair gastric mucosa	*Helicobacter pylori* rate↓, plasma motilin level↑	Feng (2015)
Randomized, controlled	Pediatric functional constipation	Emulsion (<1 year: 5 g bid; 1∼2 years: 10 g bid, >7 years: 30 g bid), 28 days (n = 40)	Negative group: diet and exercise training (n = 40)	Improve clinical symptoms	Symptoms recover rate↑, interval between defecations↓	Shi and Xuan (2020)
Randomized, controlled	Irritable bowel syndrome	Emulsion (25 g bid), 8 weeks (n = 30)	Positive group: pinaverium bromide (50 mg tid), 8 weeks (n = 15)	Improve clinical symptoms	Symptoms recover rate↑, BBS↓	Zhou and Yan (2006)
Randomized, controlled	Functional dyspepsia	Emulsion plus bifidobacterium triple viable bacteria; emulsion (25 g bid), 2 weeks; bifidobacterium triple viable bacteria (2∼5 years: 1 tablet bid; >5 years: 2 tablets bid) (n = 35)	Positive group: bifidobacterium triple viable bacteria (2∼5 years: 1 tablet bid; >5 years: 2 tablets bid), 2 weeks (n = 35)	Shorten symptom resolution time and improve gastrointestinal function	CGRP↓, CRF↓, LEP↑	Ting and Ping (2023)

## 8 Vivo studies

Studying the effects of pharmacological interventions in animal disease models is an essential scientific means in modern medicine to understand disease prevention and control laws. We have summarized ten relevant animal studies (Xing et al., 2002; Xu et al., 2007; Li R. J. et al., 2014; Liu et al., 2015; Xiao, 2017; Zhang et al., 2017; XiaoFeng, 2020; Yuan et al., 2021; Qin Q. et al., 2024; YanChao and WanXing, 2024; Yuan et al., 2024) to elucidate the preventive and therapeutic effects of sea buckthorn on digestive diseases and to provide evidence suppporting the use of sea buckthorn preparations in the daily prevention, early intervention, and clinical treatment of digestive diseases. Ulcerative colitis (UC) is characterized by chronic inflammation and ulceration of the intestinal inner lining, resulting in various symptoms (Ordás et al., 2012). While the exact mechanisms that cause the development of ulcerative colitis remain unknown, research has identified that the pathogenesis involves the release of several pro-inflammatory cytokines, including TNF-α, IL-1β, IL-6, and IL-17, which significantly drive the inflammatory response (Lee et al., 2018). In animal models of colitis, we found that sea buckthorn polysaccharides improved disease activity index, colon length, and intestinal barrier permeability (Yuan et al., 2024). Sea buckthorn polysaccharides may also reduce inflammation, oxidative stress, and intestinal barrier damage associated with colitis (Qin Q. et al., 2024). Specifically, sea buckthorn polysaccharides can inhibit the production of several inflammatory cytokines, including IL-6, IL-1β, TNF-α, and IL-17F, closely related to the downregulation of the NF-κB pathway (Yuan et al., 2024). Recent studies indicate that patients with ulcerative colitis exhibit a disruption in the gut microbiota, characterized by a significant reduction in short-chain fatty acid (SCFA)-producing bacterial species (Wang Y. et al., 2018). Sequencing analysis of intestinal flora suggests sea buckthorn polysaccharides can significantly increase microbial metabolites SCFAs and BAs to correct dysbiosis in DSS-induced colitis in mice (Yuan et al., 2024). Acute liver failure is a rare but life-threatening critical illness that most commonly affects previously healthy adults in their 30s and presents unique clinical challenges (Bernal and Wendon, 2013). It has been well documented that TLR4 signaling plays an essential role in the pathogenesis of liver injury; downregulation of TLR4 could significantly decrease hepatic c-Jun and NF-κB expression and thus decrease TNF-α levels (Ben Ari et al., 2012). Sea buckthorn possesses anti-inflammatory activity that reduces TLR4 expression to protect against LPS/d-GalN-induced liver injury (Liu et al., 2015; Zhang et al., 2017; YanChao and WanXing, 2024). The gastric mucosa (GM) is the first barrier and vital interface in the stomach that protects the host from the hydrochloric acid in gastric juice and defends against exogenous insults to the gastric tissues (Deng et al., 2023). Gastric mucosal injury is a chronic injury characterized by altered cell differentiation and is considered a precancerous lesion associated with gastric cancer (Jia et al., 2023). Existing animal models of gastric mucosal damage are mature and are mainly induced by water immersion stress, acetic acid, and ethanol. The study found that sea buckthorn extract is essential in healing acetic acid-induced gastric lesions, possibly by accelerating mucosal repair (Xu et al., 2007). The protective effect of sea buckthorn extract on the gastric mucosa was also observed in two other models of gastric mucosal injury (Xing et al., 2002; XiaoFeng, 2020). In addition to treating digestive diseases, sea buckthorn can prevent adverse medication reactions. Cisplatin-induced nausea and vomiting (CINV) remains the main problem for cancer patients in the process of oncological treatment; approximately half of cancer patients experience nausea or vomiting, either because of chemotherapy or the cancer itself (Shin et al., 2022). The study found that sea buckthorn extract prevented cisplatin-induced vomiting in rats. This may be due to its role in increasing peripheral and central OXA and the expression of OX1R in the hypothalamus and brainstem (Yuan et al., 2021). Overall, sea buckthorn has substantial health benefits, such as anti-inflammatory, intestinal barrier protection, intestinal flora balance, and the prevention of drug side effects. This suggests that supplementation incorporating sea buckthorn-related preparations in the daily diet may be a new strategy for preventing and treating digestive diseases. Further details of the animal-level experiments can be found in Table 4.

**TABLE 4:** *In vitro* experiment of sea buckthorn treatment of digestive diseases.

Disease model	Animal and molding method	Sea buckthorn group (n)	Control group (n)	Effect	Mechanism	Ref.
Colitis	C57BL/6 male mice, 3% DSS qd po for 7 days	Polysaccharides (0.2 mL*2%/day), po, 60 days (n = 12)	Positive group: synbiotics (0.2mL1*10^9^ CFU/mL), po, 60 days (n = 12)	Ameliorated disease activity index, colon length, and intestinal barrier permeability in mice	IL-6↓, IL-1β↓, TNF-α↓, IL-17F↓, IL-10↑, TGF-β↑, Foxp3↑	Yuan et al. (2024)
Colitis	C57BL/6 male mice, 2.5% DSS qd po for 7 days	Polysaccharides (0.2 mL*200 mg/kg/day), po, 21 days (n = 10)	Negative group: sterile saline (0.2 mL), po, 21 days (n = 10)	Reduces inflammation, oxidative stress, and intestinal barrier damage associated with colitis	SCFA↑, BAs↑	Qin Q. et al. (2024)
Liver failure	C57BL/6 male mice, LPS (50 μg /kg) plus d-GalN (300 mg/kg) once	Polysaccharides (50, 100, 200 mg/kg/day), po, 14 days (n = 8)	Positive group: dexamethasone (10 mg/kg), ip, once (n = 8)	Prevention of acute live injury	ALT↓, AST↓, TNF-α↓, IL1β↓, MDA↓, SOD↓, TLR4↓, p-JNK↓, NF-Κb↓	Liu et al. (2015)
Gastric ulcer	Sprague-Dawley rats, water immersion stress	Seed or pulp oils (3.5, 7 mL/kg/day), po, 7 days (n = 6)	Positive group: cimetidine (80 mg/kg/d), po, 7 days (n = 6)	Preventive and curative effects against experimental gastric ulcers	Index of pylorus ligation-induced gastric ulcer↓	Xing et al. (2002)
Cisplatin induced nausea and vomiting	Wistar rats, cisplatin (6 mg/kg) ip once	Seed oil plus ondansetron: seed oil (0.850, 1.675, 3.350 g/kg/day), po, 6 days; ondansetron 2 mg/kg/d, 6 days (n = 16)	Positive group: ondansetron (2 mg/kg/d), 6 days (n = 16)	Prevention of CINV	OXA↑, OX1R↑	Yuan et al. (2021)
Esophageal precancerous	Kun Ming male mice, 4NQO 0.1 mg/mL for 14 weeks	Dry emulsion (144 mg/day), 10 weeks (n = 45)	Positive group: all-trans retinoic acid (0.0865 mg/d), 10 weeks (n = 45)	Slow down the progression of esophageal precancerous	Cancer rate↓	Xiao (2017)
Hepatotoxicity	C57BL/6 male mice, CCl4 5 mL/kg ip once	Polysaccharides (50, 100, 200 mg/kg/day) po, 14 days (n = 8)	Negative group: distilled water, po, 14 days (n = 8)	Preventing CCl4 induced hepatotoxicity	ALT↓, AST↓, TBIL↓, PALB↑, SOD↑, GSH-Px↑, MDA↓	Zhang et al. (2017)
Gastric ulcer	Wistar male rats, 50% acetic acid 0.04 mL (v/v) laparotomy injected	Procyanidins (50, 100, 150 mg/kg/day), 14 days (n = 16)	Positive group: ranitidine (30 mg/kg), po, 14 days (n = 16)	Acceleration of the mucosal repair	UI↓, EGFR↑, EGF↑, PCNA↑	Xu et al. (2007)
Liver injury	Kun Ming male mice, LPS 10 mg/kg	Polysaccharides (50, 100, 200 mg/kg/day), 14 days (n = 8)	Negative group: saline, po, 14 days (n = 8)	Effectively inhibit lipopolysaccharide-induced liver injury	IL-1β↓, IL-6↓, TNF-α↓, NF-κB↓, p65↓	YanChao and WanXing (2024)
Gastric mucosal damage	Sprague Dawley rats, 75% ethanol 1 mL/100 g	Sterol (100, 200, 400 mg/kg/day), 7 days, (n = 16)	Positive group: ranitidine (50 mg/kg), po, 7 days, (n = 16)	Reduce ethanol induced gastric mucosal damage	SOD↑, MDA↓, GSH-Px↑	XiaoFeng (2020)

## 9 Vitro studies

**FIGURE 5:**
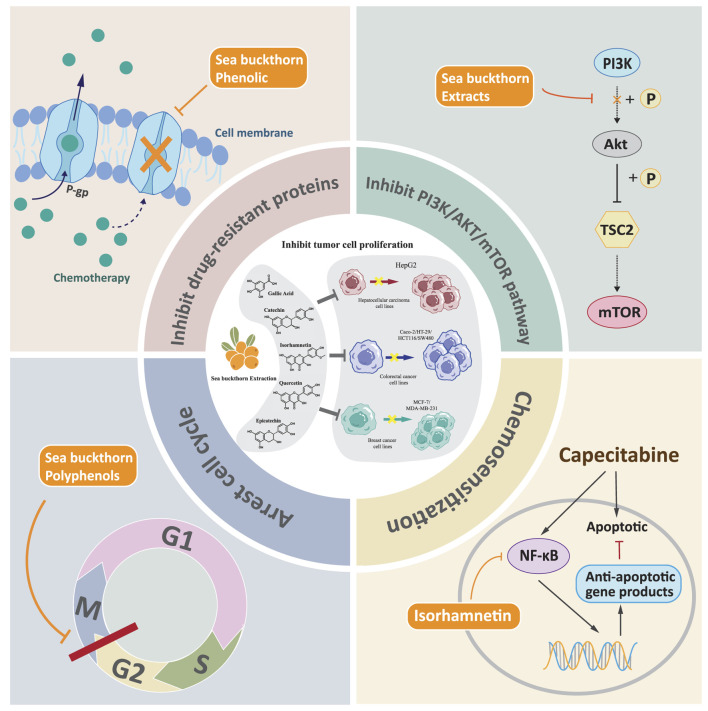
Mechanisms of Sea buckthorn anti-digestive cancer.

The above vivo experimental evidence summarizes the beneficial influence of sea buckthorn on digestive diseases at the level of the overall functioning of the organism. To further understand the mechanism of action of sea buckthorn against digestive diseases at the molecular and cellular level, we reviewed and summarized the relevant *in vitro* experiments. Current *in vitro* studies on sea buckthorn primarily focus on inhibiting cancer cells. Sea buckthorn regulates classical signaling pathways such as cell cycle PI3K/AKT, thereby suppressing the development and spread of gastric cancer by inhibiting cell proliferation, protecting the intestinal barrier, and enhancing the anticancer effects of chemotherapeutic drugs. The sustained proliferative ability of cells is an integral part of cancer, manifested by altered expression and activity of cell cycle-related proteins (Feitelson et al., 2015). Studies have shown that sea buckthorn and its active metabolites can inhibit the proliferation of cancer cells through PI3K/AKT and other signaling pathways. Sea buckthorn extract contains many bioactive metabolites with anticancer properties; the study found that it could suppress the proliferation of liver cancer HepG2 and colon cancer Caco-2 cells (Grey et al., 2010). Isorhamnetin has been found to inhibit three human colorectal cancer (CRC) cell lines, namely HT-29, HCT116, and SW480. This metabolite induces cell cycle arrest at the G2/M phase and suppresses cell proliferation by inhibiting the PI3K-Akt-mTOR pathway (Li C. et al., 2014). Additionally, Isorhamnetin enhances the anti-tumor effects of capecitabine by negatively regulating the NF-κB signaling cascade in gastric cancer (Manu et al., 2015). The phosphatidylinositol 3-kinase (PI3K)/Akt pathway plays a crucial role in various cellular processes and is aberrantly activated in cancers, contributing to the occurrence and progression of tumors (He et al., 2021). Studies have confirmed that sea buckthorn phenolic intervention can significantly reduce the levels of MRP and Pgp to inhibit the activity of HepG2, MCF-7, MDA-MB-231, and Caco-2 cell proliferation (Guo et al., 2017a). Multidrug resistance proteins can mediate cancer multidrug resistance by expelling various chemotherapeutic agents or their metabolites from tumor cells (Wang et al., 2021). Multidrug resistance (MDR), often associated with the overexpression of P-gp, has been implicated as a significant obstacle to effective chemotherapy for cancer, parasitic diseases, AIDS, and other diseases (Li et al., 2010). Active metabolites such as sea buckthorn essential oils and polyphenols also inhibit cell proliferation, and further experimental details are given in Table 5. The mechanisms of Sea buckthorn anti-digestive cancer are illustrated in Figure 5.

**TABLE 5:** *In vitro* experiment of sea buckthorn treatment of digestive diseases.

Disease model	Cell type	Sea buckthorn group	Control group	Effect	Mechanism	Ref.
Liver cancer	Hep G2	Extracts (0.25%∼2%), 48 h	Positive group: ursolic acid (80 μm ), 48 h	Inhibited cell proliferation	—	Grey et al. (2010)
Colon cancer	Caco-2	Extracts (0.25%∼2%), 24 h	Positive group: ursolic acid (80 μm ), 24 h	Inhibited cell proliferation	—	Grey et al. (2010)
Colorectal cancer	HT-29, HCT116 and SW480	Isorhamnetin (0, 40, 60, 80, 100 μm ), 24, 48, 72 h	Negative group: untreated cell	Induced G2/M phase cell cycle arrest	PI3K/AKT↓	Li et al. (2014a)
Gastric Cancer	MKN-45	Isorhamnetin (0, 2.5, 5, 10, 200, 40 μm ) for 48 h	Negative group: untreated cell	Inhibited cell proliferation	PI3K-AKT-mTOR↓	Li et al. (2021a)
Liver cancer	Hep G2	Phenolic	Negative group: untreated cell	Inhibited cell proliferation	MRP2↓, Pgp↓	Guo et al. (2017a)
Colon cancer	Caco-2	Phenolic	Negative group: untreated cell	Inhibited cell proliferation	MRP2↓, Pgp↓	Guo et al. (2017a)
Colorectal adenocarcinoma	HT-29, Caco-2	Essential Oils (5, 10, 25, 50, 75 μg/ml ), 48 h	Negative group: untreated cell	Inhibited cell proliferation	—	Dolghi et al. (2021)
Gastric cancer	SNU-5, SNU-16, MKN-45	Isorhamnetin (10 μm ), 72 h	Negative group: untreated cell	Induced apoptotic	NF-κB↓	Manu et al. (2015)
Colon cancer	HCT116, HT29, FHC	Polyphenols (0, 20, 40, 80 μg/ml )	Negative group: untreated cell	Induced G1 phase cell cycle arrest	Cyclin E↓	Wu et al. (2021)

## 10 Safety and toxicity studies

Sea buckthorn is a food with both medicinal and edible properties. With the growing use of sea buckthorn in medicinal and dietary supplements worldwide, it is essential to evaluate its safety and toxicity in order to regulate products that contain sea buckthorn. Currently, most safety and toxicity assessments of sea buckthorn focus on its oils and extracts. In a 2-week acute toxicity study, mice that were administered 20 mL/kg of sea buckthorn oil displayed no adverse reactions. Similarly, in a 90-day chronic toxicity study, rats given 10 mL/kg of sea buckthorn oil also showed no adverse effects (Zhao et al., 2017). In the teratogenicity study, pregnant rats were administered sea buckthorn oil at doses up to 4.68 g/kg starting on gestation day 16, with no treatment-related maternal toxicity or embryotoxicity observed. The findings from the genotoxicity studies indicated that SB oil showed no mutagenic activity in histidine-dependent strains of *Salmonella typhimurium*. Furthermore, SB oil did not significantly affect sperm morphology or the frequency of micronuclei in polychromatic erythrocytes in mice (Wen et al., 2020). Furthermore, research on rat burn models has demonstrated that sea buckthorn oil shows no toxicity or side effects related to wounds (Upadhyay et al., 2009). The 90-day safety study of aqueous sea buckthorn extract at a dose of 100 mg/kg body weight per day in rats showed no adverse effects on mean body weight, organ-to-body weight ratio, histological, hematological, or biochemical parameters (Tulsawani, 2010). Sea buckthorn is considered safe for consumption in food and medicine. Some studies have reported potential adverse gastrointestinal symptoms experienced by 11 participants in the SB group and 4 participants in the placebo group, respectively (*P = 0.24*) (Larmo et al., 2014). A case report study suggests that consuming 100 g of sea buckthorn syrup daily for 6 months may result in a harmless but noticeable yellow-orange skin discoloration (Grad et al., 2012). Current evidence suggests that sea buckthorn oil and extracts are generally safe; however, some studies are outdated, and research on sea buckthorn extracts is still limited. Further studies on the safety and toxicity of sea buckthorn are necessary.

## 11 Clinical application challenges

Clinical applications of sea buckthorn may encounter challenges such as regulatory policies, bioavailability, dosage standardization, and potential drug interactions. Regulations and standards for sea buckthorn products can differ by region and purpose. Adhering to safety regulations and meeting quality standards is crucial for sea buckthorn product development. Currently, sea buckthorn products, whether taken orally or applied topically, are not approved as prescription medications. Sea buckthorn seed oil and fruit extract are registered with the Food and Drug Administration (FDA) using UNII identifiers, such as UNII: T53SBG6741. This system is only designed for tracking substances, not for regulatory approval. Furthermore, the FDA does not specifically approve or endorse sea buckthorn as a dietary supplement or treatment for any disease. Sea buckthorn is sold as a dietary supplement, but claims about its ability to treat or prevent diseases are not approved by the FDA. In China, sea buckthorn is classified as food with medicinal and edible properties, allowing for its use in both food and medicinal contexts (Teng et al., 2024). The European Union regulates sea buckthorn leaves as a food metabolite under “novel food” regulations, which require specific safety assessments (Novel Food status Catalogue - European Commission, 2023). In conclusion, sea buckthorn has the potential to be used as a dietary supplement. However, it would be inappropriate to promote its pharmacological effects, particularly in the United States and Europe. Sea buckthorn is more commonly used in Chinese medicine because it is included in the Pharmacopoeia.

Sea buckthorn is abundant in flavonoids, carotenoids, fatty acids, and polysaccharides, which provide it with various pharmacological activities, but also lead to low bioavailability challenges. Sea buckthorn flavonoids are abundant and beneficial; however, they often have poor water solubility, which can hinder their absorption and bioavailability, as well as cause instability in the gastrointestinal tract and rapid metabolic clearance (Sheng et al., 2025). Utilizing phospholipid complexes may improve the absorption of flavonoids (Taldaev et al., 2025). Carotenoids, with their lipophilic nature, require dietary fat for efficient absorption from the digestive tract (Moran et al., 2018). The solution is to use nano emulsions or liposomes to enhance the absorption of carotenoids and oils (Mansur et al., 2020). Sea buckthorn polysaccharides also face challenges due to their large molecular size and low intestinal permeability (Xie et al., 2023). Research indicates that the bioavailability of polysaccharides can be effectively improved by developing appropriate drug delivery systems (DDS) for them (Li et al., 2017). Low bioavailability is a significant factor limiting the clinical application of sea buckthorn. This bioavailability can be enhanced through modifications in dosage forms and other methods.

There is still a lack of formal regulatory documents regarding standardized dosages of sea buckthorn. The variations in sea buckthorn’s active metabolite content across different regions and its diverse uses have resulted in a dosage that remains unstandardized. The standard dosage of sea buckthorn for medicinal purposes is 3∼10 g, according to the Chinese Pharmacopoeia. Empirical healers have traditionally recommended a daily dose of approximately 20 g of sea buckthorn fruit in ethnic medicine (Grad et al., 2012). Some websites related to drugs list standardized dosages of sea buckthorn, but these have not been accurately verified (Sea Buckthorn Uses, Benefits and Dosage, 2024). Health Canada’s Natural Health Products Database lists sea buckthorn oil as an approved metabolite, generally recommending a daily dosage of 1 g (Product information, 2024). Further research is needed to determine the standard dosage of sea buckthorn for medicinal use.

While no severe side effects of sea buckthorn have been reported, it is important to consider possible drug interactions when starting it alongside other medications. Sea buckthorn may decrease platelet aggregation (Sławińska et al., 2024), potentially increasing bleeding risks, especially when taken with anticoagulants like warfarin or aspirin. Sea buckthorn may enhance the hypoglycemic effects of diabetes medications (Ren et al., 2021), increasing the risk of hypoglycemia when used alongside these drugs. Sea buckthorn may enhance antihypertensive effects (Vashishtha et al., 2017), potentially leading to dangerously low blood pressure. The use of high doses of vitamin C is generally safe within therapeutic limits, but there are potential risks, such as kidney-related diseases and inaccuracies in laboratory tests (Yanase et al., 2020). Due to the high vitamin C content in sea buckthorn, its use should be carefully considered for certain patients and specific situations. In conclusion, due to the potential effects of sea buckthorn on blood glucose levels, blood pressure, and platelet function, the concurrent use of sea buckthorn and products containing it should be avoided when taking related medications.

## 12 Limitations and future research priorities

We conducted a systematic review of sea buckthorn applications in digestive system diseases, focusing on clinical studies, *in vivo* studies, *in vitro* studies, safety and toxicity studies, and potential challenges for clinical application. Our research shows that sea buckthorn has significant potential for treating digestive system diseases. However, it is important to recognize that many issues remain in the current research on sea buckthorn. Clinical studies on sea buckthorn treatment for digestive diseases reveal key issues: the number of studies is insufficient, research is somewhat outdated, and study designs lack rigor. The primary reason for the aforementioned issue is the neglect of sea buckthorn as a treatment for digestive disorders and its effectiveness. Therefore, conducting additional clinical studies on the therapeutic effects of sea buckthorn for digestive system diseases should be a priority for future research. The quantity and quality of *in vivo* animal studies on sea buckthorn are greater than those of clinical trials; however, current *in vivo* research lacks a focus on tumors of the digestive system. A thorough analysis of clinical and *in vivo* studies on sea buckthorn’s effects on digestive system disorders shows that its main therapeutic benefits include reducing inflammation, regulating functional disorders, and alleviating adverse reactions caused by related medications. However, *in vitro* studies have shown that sea buckthorn exhibits great therapeutic effects against digestive system tumors; however, it has consistently failed to advance to *in vivo* research stages. The primary reasons for this issue are the stability of sea buckthorn’s metabolism within the vivo and its ability to effectively distribute within tumor tissues. Structural modification of natural products may serve as a significant approach for discovering compounds with potential anticancer activity (Zhang X. et al., 2024). While toxicity and safety evaluations suggest that sea buckthorn has a relatively high margin of safety, caution is still advised regarding its potential interactions with other medications. In terms of clinical application, the industrialization of sea buckthorn faces several challenges. Currently, sea buckthorn is primarily positioned as a dietary supplement, and its use as a pharmaceutical still carries significant regulatory risks. Additionally, determining the optimal dosage and bioavailability of sea buckthorn are critical issues that must be addressed for its successful industrial application in the future.

## 13 Conclusion

The gastrointestinal tract is an essential life support system that performs several vital physiological functions, including digestion, absorption, and metabolism of nutrients from ingested food (Wang Y. et al., 2023). Digestive diseases comprise a wide range of conditions that affect the gastrointestinal tract and significantly impact public health. They are also a major cause of healthcare utilization and expenditure (Peery et al., 2022). Sea buckthorn is a traditional plant with an extensive history of use in both medicine and food, packed with various bioactive metabolites. It has shown great potential for extensive development in food and medicine to prevent and treat digestive diseases due to its diverse physiological functions, such as anti-inflammatory, antioxidant, immune regulatory, and cytotoxic effects on cancer cells. In recent years, numerous scientists have conducted comprehensive research on identifying, extracting, and understanding the functional properties of the bioactive metabolites in sea buckthorn. This article summarizes the clinical, animal, and *in vitro* evidence, reviewing the role of sea buckthorn and its active metabolites in preventing and treating digestive diseases. Sea buckthorn has been found to intervene in chronic gastritis, alleviate liver injury and nonalcoholic fatty liver, treat functional constipation and irritable bowel syndrome, and effectively prevent digestive diseases. It achieves this by suppressing inflammation and oxidative stress, protecting intestinal barrier function, restoring immune balance, and regulating intestinal flora. Additionally, Sea buckthorn can directly intervene in digestive cancers such as liver, colon, and gastric cancer by regulating MPR2, Pgp, mTOR, and other signaling pathways.

Our study helps digestive disease researchers take a more holistic view of sea buckthorn’s importance, which could help develop drugs and foods to improve digestive diseases. In the future, conducting in-depth investigations into the mechanisms of action to better apply sea buckthorn in food and medicine production will be essential. It is believed that more potent drugs can be discovered from sea buckthorn shortly for treating digestive diseases, reducing the medical burden of patients, and improving their quality of life.
